# High frequency of BRAF V600E mutation in Iranian population ameloblastomas

**DOI:** 10.4317/medoral.23519

**Published:** 2020-05-10

**Authors:** Samira Derakhshan, Pouyan Aminishakib, Abbas Karimi, Hiva Saffar, Alireza Abdollahi, Hadis Mohammadpour, Mohammad Javad Kharazi Fard, Amirreza Memarha

**Affiliations:** 1 DDS, MSc. Oral and Maxillofacial Pathology Department, School of Dentistry, Tehran University of Medical Sciences, Iran; 2 MD, DDS, MSc. Oral and Maxillofacial Surgery Department, School of Dentistry, Shariati Hospital, Craniomaxillofacial Research Center, Tehran University of Medical Sciences, Iran; 3 MD, MSC. Associate professor of clinical and anatomical pathology. Department of Pathology, Shariati Hospital, Tehran University of Medical Sciences, Iran; 4 MD, MSC. Department of Pathology, Imam Hospital Complex, Cancer institute, Tehran University of Medical Sciences, Iran; 5 Iran National Tumor Bank, Cancer Institute, Imam Khomeini Hospital Complex, Tehran University of Medical Sciences, Iran; 6 DDS,PHD. Dental Research Center, School of Dentistry, Tehran University of Medical Sciences, Iran; 7 DDS. School of Dentistry, Tehran University of Medical Sciences, Iran

## Abstract

**Background:**

Ameloblastoma is a common locally invasive but slow-growing neoplasm of the jaws with an odontogenic origin. Association between BRAF V600E mutation and clinicopathologic features and behavior of ameloblastoma remains controversial. This study aimed to evaluate BRAF V600E gene mutation and expression of its related proteins with clinicopathologic parameters in conventional ameloblastoma.

**Material and Methods:**

50 Formalin-fixed paraffin-embedded blocks were included in this study. Immunohistochemistry was done using rabbit monoclonal BRAF V600E mutation-specific antibody VE1. Quantitative real-time polymerase chain reaction assay was used for evaluating of BRAF V600E mutation.

**Results:**

Expression of BRAF V600E antibody was Positive in 42 out of 50 cases (84%). 46 (92%) out of 50 specimens showed BRAF V600E mutation. There were 13 cases of recurrence (26%). 3 out of 4 cases with negative mutations did not show recurrence.

**Conclusions:**

We report the highest frequency (92%) of BRAF V600E mutation in ameloblastomas in the Iranian population. Although there was not a significant association between BRAF V600E‑positive immunoexpression and recurrence and clinicopathologic parameters, its high frequency could emphasize its role as a therapeutic marker in the future.

** Key words:**Conventional ameloblastoma, BRAF V600E, recurrence.

## Introduction

Ameloblastoma is a common locally invasive but slow-growing neoplasm of the jaws with odontogenic origin (1). Although ameloblastoma does not have metastatic potential, facial deformity, significant morbidity and also recurrence in more conservative approaches, occur due to surgical treatment (2). Recent molecular findings tend to result in novel insights into discovering the pathogenesis, mechanisms and treatment of ameloblastoma. Mutation in BRAF gene-valine (V) to glutamic acid (E) substitution at codon 600 is a common mutation in ameloblastoma. Heikinheimo *et al*. reported this mutation in 74% of the analyzed conventional ameloblastoma samples (3). However, the association between BRAF V600E mutation and clinicopathologic features and behavior of ameloblastoma remains controversial (4,5). Here, we analyzed the mutation of BRAF V600E by qPCR and also the expression of BRAF V600E protein by immunohistochemistry. We also studied the correlation between BRAF V600E mutation and clinicopathologic parameters of conventional ameloblastoma.

## Material and Methods

- Samples

We included 50 Formalin-fixed paraffin-embedded (FFPE) blocks obtained from the files of the Oral and Maxillofacial Pathology Department of Tehran University of Medical Sciences, and Shariati Hospital in this study. All of the cases were diagnosed as Conventional Ameloblastoma, according to the International Histological Classification of Odontogenic Tumors guidelines published by the WHO (6). Two oral pathologists reviewed all the slides and confirmed the diagnosis. We obtained the demographic data of each sample (Table 1).

- BRAF V600E immunohistochemistry

Immunohistochemistry was done on 3-µm paraffin-embedded sections according to standard procedures. Tris/EDTA buffer solution (pH 9) (Carpinteria, Dako, CA, USA) was used to antigen retrieval following in microwave in boiling point for 15min. The slides were put in room temperature for 15min to decrease the temperature. The primary antibody incubation was performed for 1h and 30min at room temperature using Rabbit monoclonal BRAF V600E mutation-specific antibody VE1 (Recombinant RabMAb, Anti-B Raf antibody [EP152Y], ABCAM, Cambridge, Code# ab33899) with a dilution ratio of 1:150. Malignant melanoma with known presence of BRAF V600E mutation was used as positive controls. The omission of the primary antibody and using phosphate-buffered saline was done as the negative control.

- Scoring of Immunohistochemistry

Two experienced oral pathologists blindly evaluated the quantity and expression of BRAF V600E mutation for each stained section and used a composite semi-quantitative IHC-scoring scale, including intensity (0-3+) combined with staining percent of neoplastic cells (0%-100%) (7). We determined cytoplasmic brown staining in tumoral cells as positive staining and single nuclear staining, faint diffuse staining or staining of single interspersed stromal inflammatory cells as negative. We Determined positive BRAF V600E-staining intensity as follows: the absence of staining [0]; mild staining appreciable only in 400× magnification [1]; moderate staining apparent in 100× magnification [2]; and intense staining apparent in 40× magnification [3]. We determined BRAF V600E immunoreactivity by stringent scoring criteria (intensity of ≥2+ and percent of ˃10%) as described (7,8).

- Detection of BRAF V600E mutation by Quantitative Real Time-PCR

Evaluation of BRAF V600E mutation was performed using quantitative real-time polymerase chain reaction (PCR) assay. DNA was extracted from FFPE tissue samples (10-μm section per sample) using the DNA extraction kit (Yekta Tajhiz, Iran and Taiwan) according to the manufacturer’s protocols. The DNA was quantified by using the NanoDrop spectrophotometer (ThermoFisher, OneC). Real-time PCR assay designed to detect the presence of the BRAF mutation (V600E) in FFPE ameloblastoma specimens by using ANA Gene PCR kit, Iran, Karaj. Validated protocols for BRAF V600 mutation detection with a limit of detection (LOD) of 1% variant allele frequency were adopted from previously published literature. The process has been checked by using BRAF internal control. BRAF positive malignant melanoma was used as the positive control. Also, one NTC (no template control) has been used for each run as the negative control.

- Statistical analysis

We used the Statistical software IBM SPSS statistics 23.0 (IBM Corporation, Armonk, NY, USA) for the analysis of the data. We used Mann-Whitney test to find a correlation between the parameters and considered the level of P-value ≤ 0.05 as statistically significant.

## Results

- Study samples

Our results showed that 29 out of 50 cases were male (58%), with a mean age of 42 years and 21 cases were female (42%) with a mean age of 46 years. Forty-four cases were in the mandible (88%), 5 cases were in the maxilla (10%), and one case showed involvement of both jaws (2%). The involvement of the mandible was rather equally on the right (28 cases, 56%) and the left (21 cases, 42%) side. One case demonstrated involvement bilaterally (2%). The follow-up duration was between 1 to 17 (average 6) years. Majority of the cases (37,74%) did not show recurrence compared to recurrent cases (n = 13, 26%). Histologically, in most of the cases follicular pattern was predominant over the other patterns (46 cases, 92%), also 2 cases (4%) showed mainly plexiform pattern, 1 case (1%) showed mainly basal cell type and one case (2%) showed both follicular and plexiform pattern equally. All the results are present in Table 1.

**Table 1:**
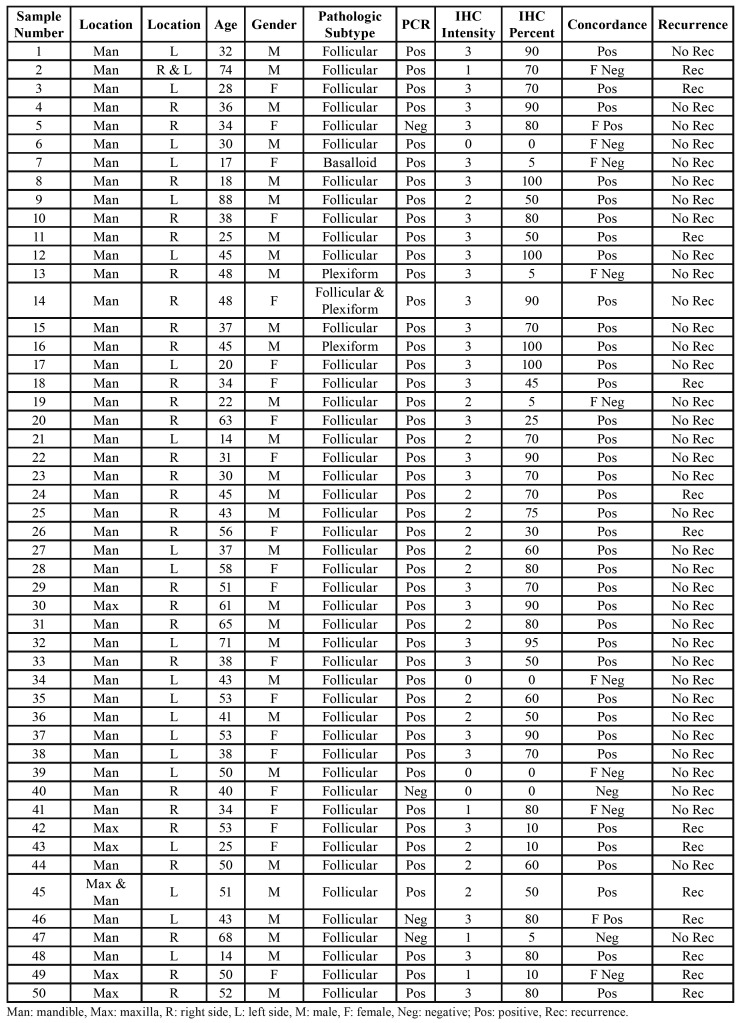
Clinical details and BRAF V600E immunohistochemical status of ameloblastoma cases.

- IHC results and molecular analysis 

**Figure 1:**
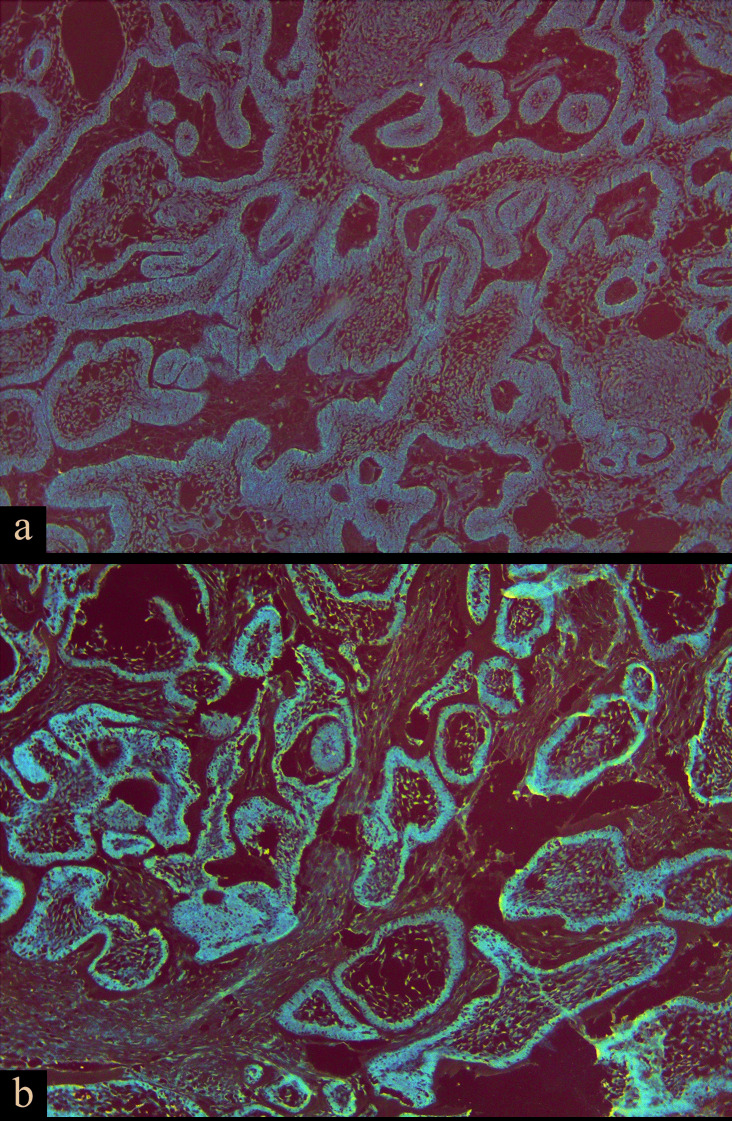
Immunohistochemistry view. a) Moderate immunostaining of tumoral cells (Plexiform pattern ×100 magnification) b) Severe immunostaining of tumoral cells (Follicular pattern ×100 magnification).

According to stringent scoring criteria (intensity of ≥2+ and percent of ˃10%), we observed positive BRAF V600E immunostaining in 42 samples (84%) (Fig. 1). 8 cases (16%) showed negative BRAFV600E immunostaining. In cases with recurrence, 11 cases showed positive immunostaining, and 2 cases showed negative immunostaining. There was no correlation between BRAF V600E immunostaining and clinicopathologic criteria (P-value higher than 0.05).

The BRAF V600E mutation was detected in 46 cases (92%) out of 50 samples. Four negative BRAF V600E mutations were in the mandible and demonstrated a mainly follicular histopathologic pattern. Clinical characteristics and molecular analysis of the four cases with negative BRAF V600E mutation are present in Table 2. In cases with recurrence, 12 of them showed a positive molecular result, and 1 case showed a negative molecular result. There was no correlation between BRAF V600E mutation and clinicopathologic criteria (P-value higher than 0.05). Recurrence was significantly associated with the occurrence of ameloblastoma in the maxilla.

**Table 2:**
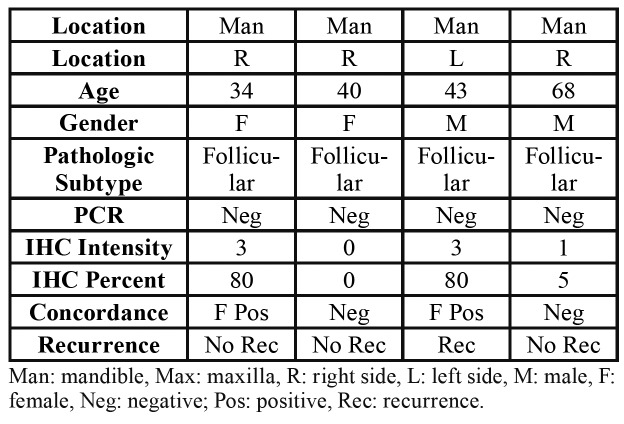
Clinical data of the four ameloblastoma cases with negative BRAF V600E mutation results.

## Discussion

Ameloblastoma is a benign slow-growing odontogenic neoplasm of the jaws which accounts for 10% of all maxillary and mandibular neoplasms (9,10).

The high recurrence rate of ameloblastoma after conservative therapy (55-90%) is a noticeable issue (9). On the other hand, functional and psychological impairments and aesthetic deformities after radical surgical treatment, especially in large ameloblastomas, are serious issues (9). It seems that a novel treatment approach may be helpful to avoid extensive surgical treatments. In 2014, several studies reported the mutation of BRAF V 600E in 40-80% of ameloblastomas (2,4,11,12). In the present study, we reported the highest frequency of BRAF V 600E (92%), which is reported in the literature in the Iranian population mutation ameloblastomas.

Different authors reported alterations in BRAF mutation frequency for ameloblastoma. Sweeney *et al*. reported the mutation of BRAF V 600E in 46% of their cases (12). Brown *et al*. and Kurppa *et al*. found BRAF V600E mutation in 62% and 63% of their cases, respectively (2,4). In 2015, one study reported by Diniz described the BRAF mutation in 82 % of their samples (11).

Gultekin *et al*. in a recent study reported the BRAF V 600E mutation in 60% of patients which was the most prevalent mutation (13). To the best of our knowledge, we report the highest frequency of BRAF V600E mutation in ameloblastomas which is reported yet as 82% by Diniz (11). There is one previous study about the BRAF V600E mutation in Iranian patients with ameloblastoma. Soltani *et al*. reported this mutation in 63% (12 of 19) of cases in a cohort study (14).

Although some papers described the correlation of BRAF mutation and clinicopathologic features such as age or anatomical location (4,13,15,16), despite the high frequency of the mutation, we did not find any significant association between clinical and histopathological findings and BRAF V600E mutation.

The correlation between BRAF V600E mutation and aggressiveness of ameloblastoma remains conflicting. Some studies described higher disease-free survival in ameloblastomas with BRAF V600E mutation (4,12). On the other hand, Fregnani *et al*. reported poor disease-free survival and more aggressive tumor for positive BRAF V 600E mutation (5). Some studies showed a lower risk of recurrence in patients with BRAF V 600E mutation (13,16), while Shirsat *et al*. described a significant association of positive immune-expression of BRAF V600E with recurrence (17). In this study, we did not find any association between BRAF V600E mutation and recurrence. Although we found a higher recurrence rate in maxillary tumors, this higher recurrence rate may also be due to treatment limitations based on limited access to safe margins.

There are some *in vitro* studies that have described the sensitivity of BRAF inhibitors in ameloblastoma cells with BRAF V600E mutation (4,12). Besides, three cases with BRAF V600E mutation showed a successful response to BRAF inhibitors (18-20). It seems that BRAF inhibitors can have clinical benefits and responses in recurrent and metastatic ameloblastomas and use as neoadjuvant and/or targeted adjuvant therapy to improve the treatment outcome, especially in locally advanced ameloblastomas (21,22). Although new molecular medicine demonstrates personalized targeted therapy for ameloblastoma, it seems that based on lack of large scale clinical trials, evaluation of the wide clinical application of BRAF inhibitors has a long way. 

According to significant evidence of activating MAPK pathway in the pathogenesis of ameloblastoma (4) and since BRAF is the most prominent activator of this pathway (23,24), high frequency of BRAF mutation in ameloblastoma in Iranian population demonstrate the possibility of MAPK pathway activation in the pathogenesis of this aggressive benign tumor.

Our results did not show a correlation between BRAF V600E immunoexpression and molecular analysis of this mutation. These findings are not consistent with the previous studies of readily detecTable of BRAF V600E expression by immunohistochemistry evaluation and correlation with mutation status in ameloblastoma (25-27).

To our surprise, we detected the highest frequency of BRAF V600E mutation in our study. It seems that geographic and ethnic criteria may be possible reasons for these results and future multicentric studies are required for further evaluation and analysis of BRAF V600E mutation status and its role as a predictor or therapeutic marker.

We want to highlight the significance of our study that has a large sample size and accepTable follow-up duration in which both mandibular and maxillary ameloblastomas are included.

## Conclusions

The present study reported the highest frequency of BRAF V600E mutation in ameloblastomas (92%) compared with the previous studies till now. We highlighted the correlation between BRAF V600E immunoexpression and molecular analysis of this mutation in ameloblastomas. Since the dependency of clinicopathologic data and BRAF V600E mutation in ameloblastomas remains questionable and conflicting, further studies are required in future to explain the real relationship of this mutation with the aggressiveness of ameloblastoma.
